# The Microbiome Within a Microbe: Rethinking *Blastocystis* Biology

**DOI:** 10.1111/jeu.70056

**Published:** 2026-01-26

**Authors:** Daisy Shaw, Eleni Gentekaki, Anastasios D. Tsaousis

**Affiliations:** ^1^ Laboratory of Molecular and Evolutionary Parasitology School of Natural Sciences, University of Kent Canterbury UK; ^2^ Department of Veterinary Medicine University of Nicosia School of Veterinary Medicine Nicosia Cyprus

**Keywords:** *Blastocystis*, eukaryome, gut microbiome, holobiont, prokaryome, symbiosis, virome

## Abstract

*Blastocystis* spp., one of the most prevalent microeukaryotes in the human gut, has long puzzled researchers with its ambiguous role in health and disease. Decades‐old microscopy studies reported bacterial‐ and viral‐like particles within *Blastocystis* spp. cells, but these findings have been mainly overlooked. Comparable associations in other protozoa, such as those between *Trichomonas vaginalis* and *Mycoplasma*, as well as protozoan–virus interactions, are known to influence metabolism, immune evasion, and ecological fitness. Here, we revisit these neglected observations in *Blastocystis* spp., framing them within the holobiont concept and proposing that this protist may host its own microbial consortium. We also propose potential mechanisms, ecological implications, and modern experimental strategies—from organ‐on‐a‐chip to single‐cell multi‐omics—to rigorously test this hypothesis. Recognizing *Blastocystis* spp. as a possible “microbiome within a microbe” could transform our understanding of its biology and its place in gut microbial ecology.

## 1 Introduction

*Blastocystis* spp., a protist of controversial pathogenicity, is found in the intestinal microbiota of an estimated one billion people worldwide (Scanlan and Stensvold 2013). It is frequently reported as the most common protist in fecal samples (Tan 2008), reflecting a high global prevalence in both human and animal hosts. Despite this ubiquity, *Blastocystis* spp. remains an enigmatic member of the gut microbiome, with many aspects of its biology still poorly understood.

Most observations of *Blastocystis* spp. morphology and behavior are from in vitro visualizations of cultures, which have adapted to the laboratory environment over decades, with only a few studies examining it in an in vivo setting. Existing in vivo investigations have focused on rodents, but most examples of this have been carried out with subtypes that are not natural colonizers of these animals (Gao et al. 2024; Yason et al. 2019). If we are interested in the role that *Blastocystis* spp. plays in health and disease, specifically in humans, we may need to adapt the methods we use to observe its behavior and relationship with other microbes.

Cultivation of *Blastocystis* spp. can be achieved under both xenic and axenic conditions, yet each approach poses significant challenges (Shaw, Denoyelle, et al. 2025; Shaw, Edwards, et al. 2025). Xenic culture, in which the protist is maintained alongside co‐occurring gut microbes, is a relatively straightforward and well‐established method. The presence of facultative anaerobic bacteria in such cultures can reduce oxygen levels, allowing *Blastocystis* spp., which otherwise thrives under anaerobic conditions, to survive in microaerophilic environments. In contrast, axenic culture requires strict anoxia and has been successfully achieved for only a limited number of subtypes (Chen et al. 1997; Deng and Tan 2022; Ho et al. 1993; Zierdt et al. 1988). The difficulty in sustaining axenic cultures is generally attributed to the organism's sensitivity to oxygen (Tsaousis et al. 2012); however, another possibility is that *Blastocystis* spp. relies on interactions with other gut microbes for optimal growth and survival.

In this perspective, we revisit historical observations and draw on analogies from other protozoa to explore the hypothesis that *Blastocystis* spp. engages in endosymbiotic relationships with members of the gut microbiota. We present evidence suggesting the existence of endosymbionts in *Blastocystis*, discuss potential interactions that may be integral to its biology, and propose experimental approaches to test this idea. By reframing *Blastocystis* spp. as a potential holobiont, we highlight how this perspective could reshape our understanding of its ecological role and impact on host health.

## 2 Methods

A literature review was conducted to identify previous articles that visualized *Blastocystis* spp. via electron microscopy (EM) and/or with evidence of endosymbionts. A total of 40 articles (Table S1) were identified, and these were scanned for evidence of symbionts. Out of these articles, seven proposed symbiosis within *Blastocystis* spp., three of which showed evidence of prokaryotic symbionts; two were suggestive of engulfment of bacteria‐like symbionts; and two showed alleged viral symbionts. The types of samples imaged varied in origin, from fresh fecal samples to in vitro cultures, both xenic and axenic (Table 1). In vitro cultures ranged from freshly established to up to 3 years in liquid medium. The remaining articles showed no evidence of suggested symbionts.

**TABLE 1:** Existing literature shows endosymbionts in *Blastocystis*.

Authors	Summary	Sample origin
Zierdt and Williams (1974) *Experimental Parasitology*	Axenisation of *Blastocystis* culture with ampicillin, streptomycin and amphotericin B highlighted the anaerobic nature of *Blastocystis* and the requirement for pre‐reduced media for cultivation excluding bacteria. Stable support of bacterial‐like endosymbionts was noted under these conditions and observed via light microscopy.	Axenisation of in vitro cultures in egg medium slants, originating from human fecal material from subjects with diarrhea
Zierdt and Tan (1976a, 1976b) *Experimental Parasitology*	TEM and freeze‐etch electron microscopy showing spherical and rod‐shaped structures inside *Blastocystis* after axenisation with antibiotics.	Axenic in vitro cultures maintained in egg medium, originating from human fecal material from subjects with diarrhea
Teow et al. (1992) *Parasitology Research*	*Blastocystis* obtained from the sea snake *Lapemis hardwickii* were observed to be harboring virus‐like particles, via TEM. The particles were icosahedral in shape and roughly 30 nm in diameter.	Axenised in vitro cultures maintained in Boeck‐Drbohlav's medium, originating from sea‐snake cecum
Stenzel and Boreham (1994) *International Journal for Parasitology*	TEM observation of fresh fecal material from *Macaca macaca* and *Anas platyrhynchos* showed presence of vacuolar *Blastocystis* cells, containing bacteria‐like endosymbionts, measuring approximately 0.2 μm in length.	Duck and monkey fecal material
Suresh et al. (1994) *Parasitology Research*	Observation of adherence of bacteria to the surface coat, as well as imaging of ingestion of bacteria into the amoeboid via TEM, and breaks in the surface coat, proposed to allow bacteria to enter to cell.	Encysted in vitro cultures, originating from human fecal material
Stenzel and Boreham (1997) *International Journal for Parasitology*	Fresh simian fecal samples from *Macaca fascicularis* were examined by TEM and particles with electron‐opaque cores were observed. Some were 60 nm in diameter and other were slightly larger, at 100 nm in diameter.	Simian fecal material
Tan and Suresh (2006a, 2006b) *Parasitology Research*	SEM and TEM were employed to image the amoeboid form of *Blastocystis* and bacteria were seen to be attached to the surface coat, surrounded by pseudopodia and some enclosed by the surface coat.	In vitro cultures maintained in Jones' medium, originating from human fecal material

## 3 Results and Discussion

**FIGURE 1:**
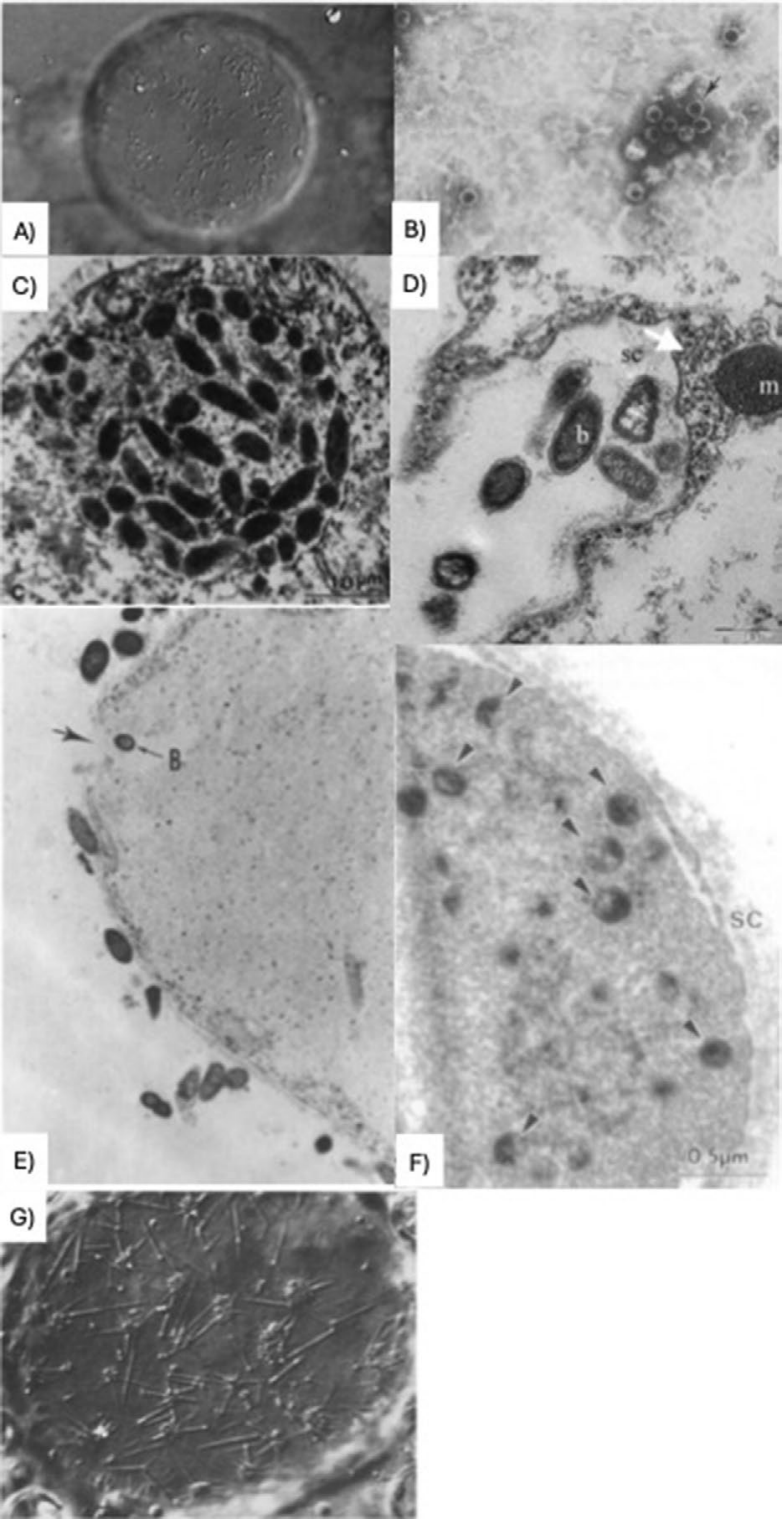
*Blastocystis* endosymbionts have been previously observed as (A) light microscopy of axenised *Blastocystis* with rods, spheres and filaments (Zierdt and Williams 1974, 240, reprinted with permission from Elsevier), (B) TEM imaging of icosahedral virus‐like particles (Teow et al. 1992, 1031, reprinted with permission from Elsevier), (C) TEM of membrane‐bound endosymbionts inside *Blastocystis* (Stenzel and Boreham 1994, 148, reprinted with permission from Elsevier), (D) TEM of *Blastocystis* pseudopodia engulfing bacteria (Tan and Suresh 2006a, 740, reprinted with permission from Springer Nature), (E) TEM of bacteria being engulfed by *Blastocystis* (Suresh et al. 1994, 332, reprinted with permission from Springer Nature), (F) TEM of hexagonal particles within the *Blastocystis* cytoplasm (Stenzel and Boreham 1997, 346, reprinted with permission from Elsevier), (G) TEM of rod‐shaped endosymbionts within the *Blastocystis* vacuole (Zierdt and Tan 1976a, 425, reprinted with permission from Elsevier).

Although rarely discussed, several historical reports suggest that prokaryotic microorganisms can reside within the vacuole of *Blastocystis* spp. (Table 1). The earliest observation can be dated back to 1974, where Zierdt and Williams reported rods and cocci within the vacuole using light microscopy (Figure 1A). In this study, they axenised a culture of *Blastocystis* spp. over the course of 6 weeks, using ampicillin, streptomycin, and amphotericin B. Failed attempts to establish axenic culture of *Blastocystis* spp. suggested that a period of adaptation to reduced bacterial presence was needed prior to axenisation, which was later achieved through gradual reduction of associated bacteria. Evidence of endosymbiosis was present in isolates from freshly collected stool samples, but was much more evident in the axenised cultures (Zierdt and Williams 1974). In 1976, Zierdt and Tan published in further detail the observation of spherical and rod‐shaped structures inside *Blastocystis* spp., using TEM and freeze‐etch electron microscopy (Figure 1G). Removal of endosymbionts was attempted using a mixture of different concentrations of rifampin, chloramphenicol, and tetracycline, but was largely unsuccessful. Concentrations of 50 μg/mL tetracycline appeared to remove the endosymbionts referred to as ‘alpha,’ but they reappeared once *Blastocystis* spp. was cultured in antibiotic‐free media. Hence, it was concluded that the endosymbionts must have remained in low abundance despite treatment. In axenic culture, numbers of bacterial‐like endosymbionts seemed to have a direct relationship with large, up to a diameter of 200 μm, *Blastocystis* spp. cells, and they were released into the medium when *Blastocystis* spp. cells lysed. However, any attempts to culture the bacterial endosymbionts separately from *Blastocystis* spp. were unsuccessful (Zierdt and Tan 1976a, 1976b). Not only have prokaryotic‐like microorganisms been observed inside this protist, but it has also been noted twice in the literature that viral‐like particles (VLPs) exist alongside *Blastocystis* spp. (Stenzel and Boreham 1997; Teow et al. 1992), suggesting that this microorganism could be hosting various endosymbionts. Teow et al. did not observe VLPs inside *Blastocystis* spp. cells themselves, but extracted a dsRNA containing fraction, corresponding to nuclear and cytoplasmic regions of the cell. When these fractions were visualized via EM, icosahedral shapes were observed (Teow et al. 1992; Figure 1B), although these were only found in a *Blastocystis* spp. isolate from the sea snake *Lapemis hardwickii* and were absent in human isolates. These findings were then supported by Stenzel and Boreham in 1997, who successfully visualized these VLPs inside the *Blastocystis* spp. cytoplasm via transmission electron microscopy (TEM) (Figure 1F). These isolates were collected from fresh fecal material of *Macaca fascicularis* monkeys, and both 30 nm icosahedral shaped particles and hexagonal particles with a larger size of up to 200 nm were seen (Stenzel and Boreham 1997). In 1994, Stenzel and Boreham used TEM to observe “bacteria‐like endosymbionts” in the form of bacilli inside both vacuolar and cyst forms of *Blastocystis* spp. (Figure 1C). The bacilli from different host organisms differed in size, with those inside *Blastocystis* spp. from duck ( *Anas platyrhynchos* ) measuring up to 1 μm in length, whilst those seen within cells from the monkey (formerly *Macaca macaca*) measuring up to 2 μm in length (Stenzel and Boreham 1994). In the same year, Suresh et al. imaged the engulfment of bacteria into the amoeboid form of *Blastocystis* spp. using TEM (Figure 1E). They also separately observed breaks in the fuzzy coat of the protist, thought to allow entry of bacteria into the cell, further supported by images of bacteria within vacuoles (Suresh et al. 1994). In 2006, Tan, Suresh et al. imaged bacteria surrounded by pseudopodia of amoeboid *Blastocystis* spp., thought to be about to undergo engulfment (Figure 1D). This was observed in *Blastocystis* spp. isolates from symptomatic (diarrhoeic) humans, and the authors suggested that pseudopodia may enable the amoeboid form of the protist to carry out phagocytosis (Tan and Suresh 2006a, 2006b). The origin of the samples in these studies ranged from fresh stool to cultured isolates, both newly established and long‐term cultures, suggesting that these endosymbionts were not lost in domesticated cultures. Notably, these endosymbionts were observed in a variety of isolates from different host organisms. More recently, a metagenomic data‐mining study identified several novel RNA viruses associated with *Blastocystis* spp. isolates spanning diverse viral families and providing *in silico* evidence for potential viral symbionts (Starrett et al. 2021). However, the source of these genomes is unclear, and consequently, the validity of the annotations is uncertain. Nonetheless, these findings support earlier microscopy‐based observations and once confirmed, could open avenues for exploring whether viral symbionts influence *Blastocystis* spp. biology and potentially alter its behavior and effect on the host, as has been demonstrated for other protozoa. Scanning electron microscopy (SEM) has provided further evidence, showing apparent endocytosis of prokaryotic microorganisms into the vacuole, where they were subsequently observed via TEM and light microscopy (Figure 1A,C–E).

Bacterial and archaeal endosymbionts are found in many protists (Husnik et al. 2021). Protozoa are well known for their ability to internalize other microorganisms, and numerous cases of endosymbiosis across the spectrum from mutualism to parasitism have been described (Dessì et al. 2019), Hence, the critical question is whether *Blastocystis* spp. truly contains endosymbionts and if this interaction benefits *Blastocystis* spp., its endosymbionts, or both. If these organisms are indeed endosymbionts, then their presence might be the underlying reason for the notorious difficulty of maintaining axenic cultures.

A useful parallel is *Trichomonas vaginalis*, which harbors *Mycoplasma* spp. endosymbionts, with infection rates ranging from 5% to 89% (Fichorova et al. 2017). This association alters *T. vaginalis* gene expression and enhances pathogenicity by increasing cytoadhesion and haemolytic activity in vitro (Ong et al. 2022). For *Mycoplasma hominis* , the intracellular niche of *Trichomonas* provides protection from both the host immune system and antibiotic treatments (Wang and Wang 1986). Viruses also play a role: *T. vaginalis* harbors *Trichomonasvirus* (Dagar et al. 2024), which influences parasite virulence by modulating cysteine protease expression (Goodman et al. 2011). Members of the *Totiviridae* family, to which *Trichomonasvirus* belongs, are common in protozoa, infecting *Giardia* and *Leishmania* (Dziallas et al. 2012), while viral symbionts in *Cryptosporidium parvum* have been linked to increased transmissibility via higher oocyst shedding in calves (Jenkins et al. 2008). Another example of an intestinal protist harboring microbial endosymbionts is *Pseudotrichonympha grassii*, which inhabits the gut of *Coptotermes formosanus* , a termite. The bacteria within this ‘triplex symbiosis system’ were found to be of the order Bacteroidales (Noda et al. 2007).

Other protozoa provide additional context. Ciliates, for example, not only harbor prokaryotic symbionts but can also serve as symbionts themselves (Dagar et al. 2024). Their large cell size makes them suitable hosts for diverse microorganisms, conferring benefits such as shared nutrient pools, detoxification of host waste, and enhanced dispersal of symbionts through host motility (Dziallas et al. 2012). The ciliate *Paramecium aurelia* has been seen to harbor Gram‐negative bacteria inside its cytoplasm. Preer et al. suggested that *P. aurelia* cells that would otherwise be sensitive to the bacterial toxins are resistant to these secretions when they are inhabited by these endosymbionts (Preer et al. 1974). *Paramecium* spp. have also been shown to host ten different species of *Holospora* spp., which have been shown to be localized to the host nucleus, below the nuclear envelope (Fokin et al. 1996). Infection with *Holospora* spp. has shown to increase the size of the *Paramecium* spp. macro‐ and micronucleus (Fokin and Görtz 2009). Symbiotic interactions are not limited to protozoa; parasitic helminths such as *Taenia* spp. and *Echinococcus* spp. illustrate parasitism on a multicellular scale, where the host derives no benefit while the parasite thrives (Adukpo 2025; Nakao et al. 2010).

**FIGURE 2:**
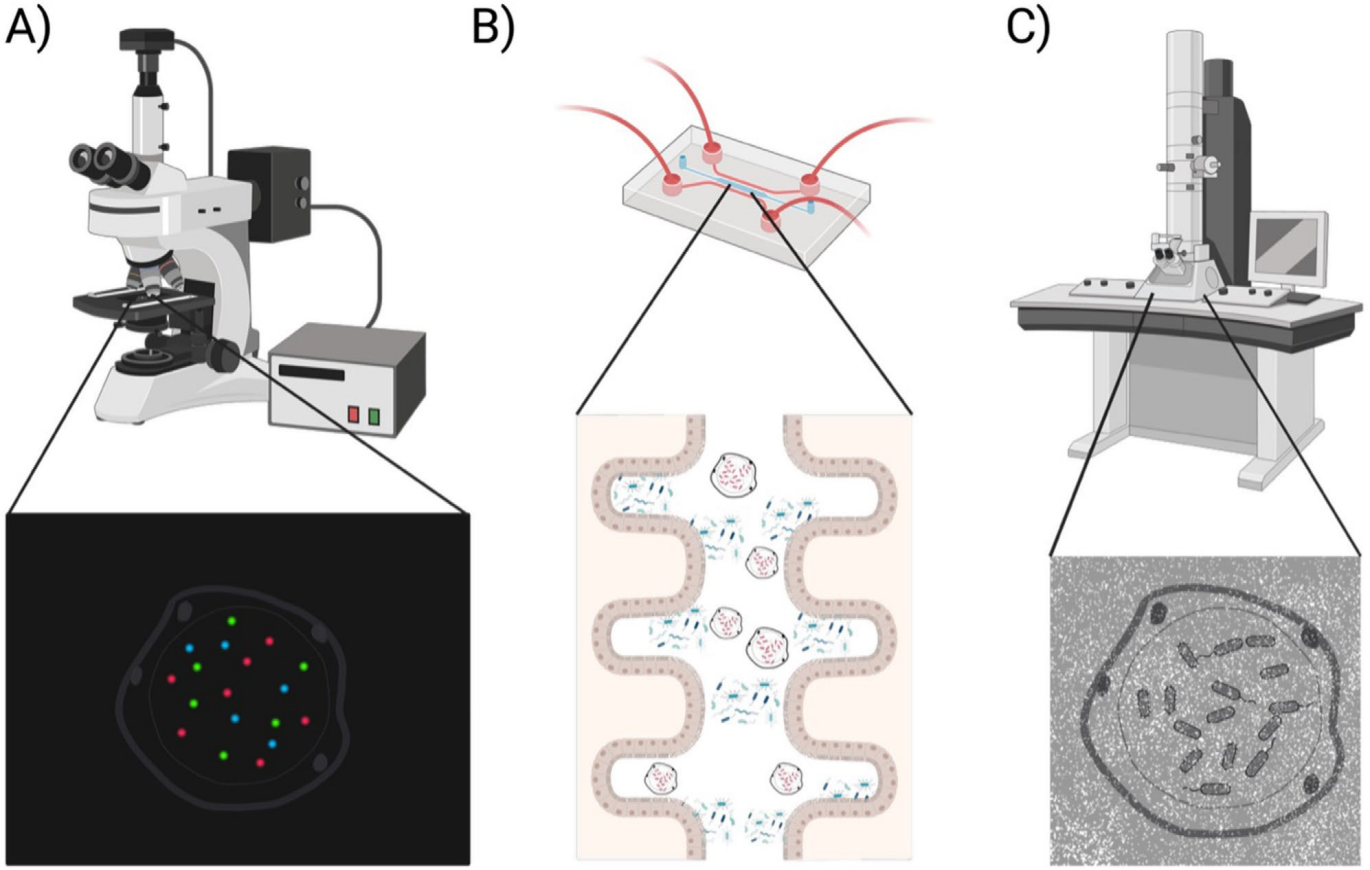
Technologies for observing symbiotic interactions include (A) fluorescence in situ hybridisation (FISH), (B) organ‐on‐a‐chip technologies and (C) transmission electron microscopy (TEM).

Taken together, these examples raise the possibility that the few reports of endosymbionts within *Blastocystis* spp. are not anomalous but fall in line with broader patterns of symbiosis in microorganisms. The challenge now is to confirm and characterize these interactions. TEM remains a powerful tool for visualizing intracellular structures, but verifying endosymbiosis requires demonstrating that engulfed microorganisms are alive and metabolically active. Fluorescence in situ hybridisation (FISH; Figure 2), using probes targeting 16S rRNA, could help distinguish viable bacterial or archaeal symbionts within *Blastocystis* spp. (Harmsen et al. 2002).

To sustain and interrogate these associations, advanced culture systems such as organ‐on‐a‐chip (OoC) offer unique opportunities (Leung et al. 2022). OoC models can recreate microphysiological conditions of the gut and allow live or time‐lapse microscopy to monitor symbiotic relationships dynamically (Buchanan and Yoon 2022; Farhang Doost and Srivastava 2024) (Figure 2). The integration of host intestinal epithelial cells would further enable the assessment of the impact of *Blastocystis* spp.–symbiont interactions on the host environment, for example, by measuring transendothelial electrical resistance (TEER) as a proxy for barrier integrity (van der Helm et al. 2016).

Complementary imaging techniques, including TEM and SEM, remain essential for high‐resolution visualization of symbiont localisation and morphology, as well as live microscopy of fresh samples. These studies suggest that endosymbionts primarily reside within the vacuole of *Blastocystis* spp., an organelle that occupies up to 90% of the cell in its vacuolar form (Tan 2008). While lipid storage (Chandrasekaran et al. 2014) and autophagic functions (Yin et al. 2010) have been proposed, the prevalence of this form in both culture and fecal samples (Tan 2004) suggests that the vacuole also serves as a niche for symbiotic partners.

As a result, several unresolved questions arise. What types of bacteria occupy the vacuole: commensals, beneficial partners, or potential pathogens? Could *Blastocystis* spp. buffer dysbiosis by sequestering bacteria during antibiotic treatment, or conversely release pathogenic species when exposed to anti‐parasitic drugs such as metronidazole, which has both anti‐protozoal and antibacterial properties? Could strict anaerobes find refuge in this highly anoxic compartment? These questions highlight the need to revisit and expand upon the early observations of endosymbionts in *Blastocystis* spp. using contemporary tools. Doing so could transform our understanding of this enigmatic protist and its ecological role within the gut microbiome.

## 4 Outlook

**FIGURE 3:**
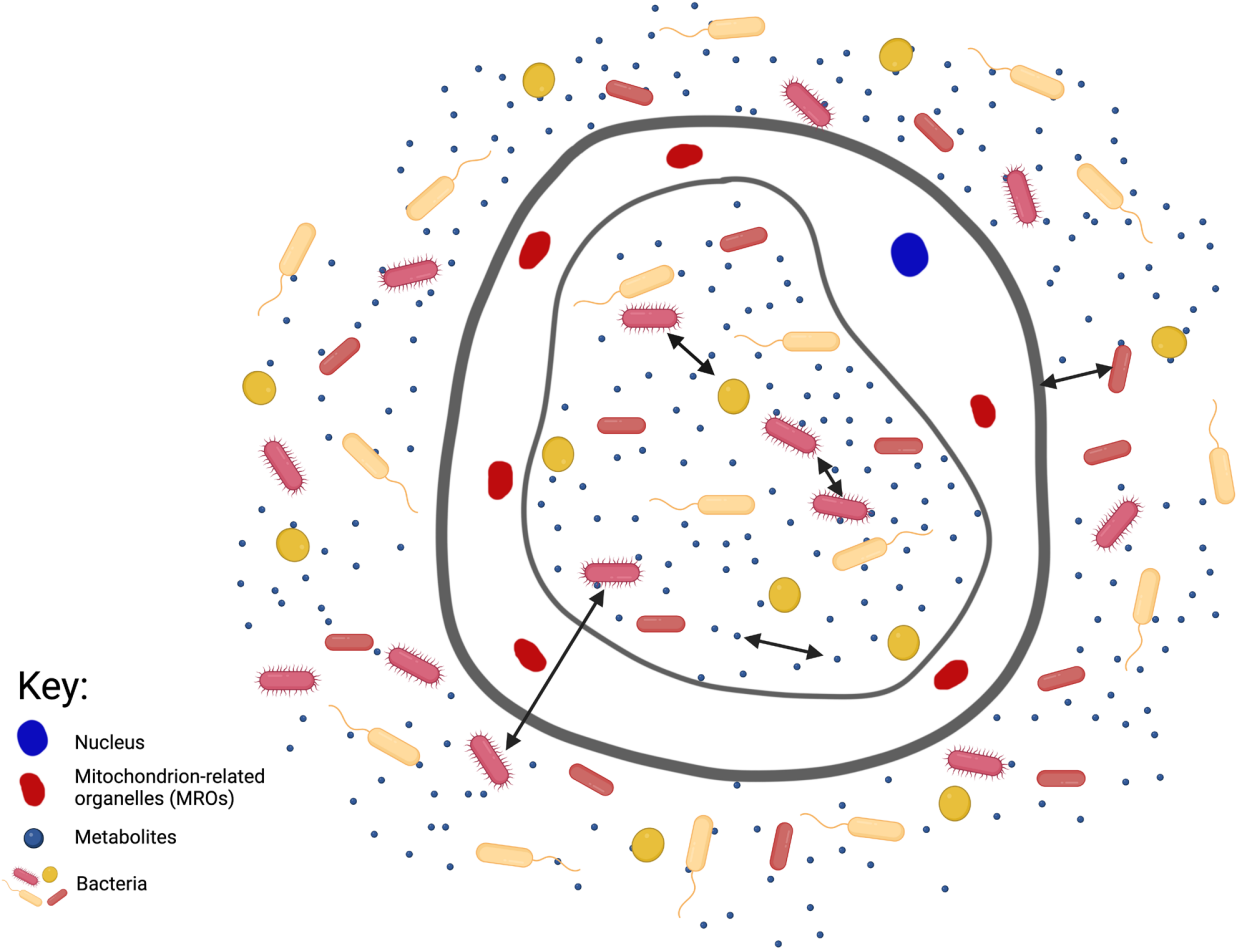
*Blastocystis* exists in a holobiotic environment. Interactions between prokaryotic members of the microbiota, excreted metabolites and *Blastocystis* itself are essential for the functioning of this microorganism and its role in health and disease.

The overlooked reports of bacterial‐ and viral‐like endosymbionts within *Blastocystis* spp. challenge the long‐standing view of this protist as a solitary gut inhabitant. If *Blastocystis* spp. functions as a holobiont (Figure 3), hosting its own microbial partners, it may profoundly alter how we interpret many aspects of its biology, persistence, and role in health and disease. The key priorities now are to (i) rigorously confirm the presence and viability of intracellular microbes using modern approaches such as FISH, single‐cell multi‐omics, and live imaging; (ii) explore the ecological and metabolic consequences of such associations through organ‐on‐a‐chip models and integrated host–microbe systems; and (iii) assess whether these symbionts modulate pathogenicity, immune interactions, or responses to antimicrobial treatment. Answering these questions could reposition *Blastocystis* spp. from a controversial commensal to a model for studying microbe–microbe–host interactions in the gut ecosystem.

## Supporting information


**Table S1:** List of previously published manuscripts describing the *Blastocystis* structural characteristics using electron microscopy.

## Data Availability

The authors have nothing to report.
