# Supplementary material for: The Microbiome Within a Microbe: Rethinking Blastocystis Biology
Source: J Eukaryot Microbiol. 2026 Jan 26;73(1):e70056. doi: 10.1111/jeu.70056 (PMC12835684; doi:10.1111/jeu.70056)
Supplement: Supplementary file 1 — Table S1: List of previously published manuscripts describing the Blastocystis structural characteristics using electron microscopy. [file JEU-73-e70056-s001.docx]

Supplementary Table 1.

| **Authors** | **Title** | **Observation** |
| --- | --- | --- |
| Tan and Zierdt, 1973 | Ultrastructure of *Blastocystis hominis* | Early observation of amoeboid, granular and vacuolar forms of *Blastocystis* via TEM. |
| Tan et al., 1974 | Freeze-etch studies of the granular and vacuolated forms of *Blastocystis hominis* | Confirmation of many features previously seen with other microscopy methods, using freeze-etch microscopy |
| Zierdt and Tan, 1976 | Ultrastructure and Light Microscope Appearance of *Blastocystis hominis* in a Patient with Enteric Disease | TEM was used to visualise *Blastocystis*, and it was noted that the nucleus had a crescent-like concentration of chromatin, endoplasmic reticulum was present, and mitochondria and vesicles were clustered to one pole of the cell. |
| Zierdt, 1986 | Cytochrome-Free Mitochondria of an Anaerobic Protozoan - *Blastocystis* hominis | TEM imaging of *Blastocystis* showed a very large central vacuole with a thin cytoplasm at its edge, containing two nuclei and a mitochondrion. |
| Mehlhorn, 1988 | *Blastocystis* hominis, Brumpt 1912: Are there different stages or species? | Two forms of cyst-like organisms observed: one with a non-granulated capsule, and the other with a granulated capsule. |
| Dunn et al., 1988 | Ultrastructural variation of *Blastocystis hominis* stocks in culture | The vacuolar and amoeboid forms of *Blastocystis* were observed with TEM. |
| Stenzel et al., 1993 | Morphology of *Blastocystis* sp. isolated from circus animals | Observation of *Blastocystis* obtained from camel, llama, bull and lion, with bacteria seen near the surface coat. |
| Stenzel et al., 1994 | Morphology of *Blastocystis* sp. from domestic birds | Observation of various morphological forms of *Blastocystis* via TEM, with many intracellular structures identified. |
| Singh et al., 1995 | Elucidation of the life cycle of the intestinal protozoan *Blastocystis* hominis | TEM comparing thin and thick-walled cysts and SEM of a ruptured cyst. |
| Yoshikawa et al., 1995 | Histochemical Detection of Carbohydrates of *Blastocystis hominis* | *Blastocystis* was stained with periodic acid methenamine silver (PA-MS) or periodic acid thiocarbohydrazide-silver proteinate (PA-TCH-SP), then visualised with TEM, where the staining could be seen on the central vacuole, Golgi apparatus and cytoplasmic vesicles. |
| Suresh et al., 1995 | Tubulovesicular elements in *Blastocystis* *hominis* from the caecum of experimentally-infected rats | Presence of tubulovesicular elements connecting to the periphery of the cytoplasm, and ribosome-like particles in *Blastocystis,* via TEM |
| Lee and Stenzel, 1999 | A survey of *Blastocystis* in domestic chickens | TEM showed a bacterial cell was embedded in the surface coat of a vacuolar *Blastocystis* cell. |
| Yoshikawa et al., 2004 | A survey of *Blastocystis* infection in anuran and urodele amphibians | *Blastocystis* isolates derived from amphibians, which did not survive in culture at 37°C, but rather 25°C, were imaged using TEM, showing dense mitochondria, and a dense central vacuole. These isolates also showed evidence of endocytic sites on the plasma membranes. |
| Yin et al., 2010 | Autophagy is involved in starvation response and cell death in *Blastocystis* | TEM observations of *Blastocystis* comparing untreated controls with cells showing evidence of autophagic-like vacuoles under conditions of amino acid starvation or rapamycin treatment. |
| Stenzel and Boreham, 1996 | *Blastocystis* hominis revisited | TEM observations of *Blastocystis* cells containing many ultrastructural features, such as lysosome-like bodies, rough endoplasmic reticulum and mitochondrion-like organelles (MLOs), and evidence of a bacterium in close association with the surface coat. |
| Yoshikawa, 1996 | Freeze-fracture Cytochemistry of Membrane Cholesterol in *Blastocystis hominis* | Freeze fracture using filipin for cholesterol visualisation showed a heterogeneous distribution of intramembrane particles, and many filipin-cholesterol complexes were seen in intramembrane particle-free areas. |
| Moe et al., 1996 | Observations on the ultrastructure and viability of the cystic stage of *Blastocystis* *hominis* from human feces | TEM showed a less electron-dense cell wall, and bacteria were found making contact with the cyst wall and fibrillar layer of some *Blastocystis* cells. |
| Stenzel et al., 1997 | Morphological differences in *Blastocystis* cysts – an indication of different species? | TEM was used to visualise cyst forms of *Blastocystis* which were measured at approximately 4 μm in diameter. Bacteria were seen to be in contact with the outer fibrillar layer of cyst forms and the surface coat of vacuolar forms. |
| Tan et al., 1997 | Survival of *Blastocystis* *hominis* clones after exposure to a cytotoxic monoclonal antibody | Immunogold TEM showed presence of epitopes recognised by monoclonal antibodies 1D5, IE7 and 4F7 |
| Duda et al., 1998 | Detection of *Blastocystis* sp. in domestic dogs and cats | Imaging showed several key cell components, such as nuclei, mitochondria, a central vacuole and bacteria nearby, but not inside the *Blastocystis.* |
| Chen et al., 1999 | *In vitro* encystation and excystation of *Blastocystis ratti* | Transmission electron microscopy showed binary fission of *Blastocystis* cysts. |
| Moe et al., 1999 | Development of *Blastocystis hominis* cysts into vacuolar forms in vitro | Development of cysts into vacuolar form were observed for after 24 hours via TEM and binary fission was observed after 12 and 18 hours. |
| Tan et al., 2001 | Do *Blastocystis* hominis colony forms undergo programmed cell death? | Presence of large gaps between cells in the colon, margination of chromatin and fragmentation into membrane enclosed structures to prevent release of lytic cell contents suggest programmed cell death. |
| Nasirudeen, et al., 2001 | Programmed cell death in a human intestinal parasite, *Blastocystis* *hominis* | Observation of morphological features associated with apoptosis-like cell death, such as condensation and fragmentation of the nucleus and release of apoptotic bodies into the extracellular space. |
| Nasirudeen and Tan, 2004 | Isolation and characterization of the mitochondrion-like organelle from *Blastocystis hominis* | Negative staining and TEM showed MLOs, with DAPI staining confirm presence of DNA in these organelles. |
| Nasirudeen et al., 2004 | Metronidazole induces programmed cell death in the protozoan parasite *Blastocystis hominis* | TEM showed that exposure to metronidazole caused morphological changes associated with programmed cell death, such as reduced cytoplasmic volume and condensation of the nucleus. |
| Tan and Nasirudeen, 2005 | Protozoan programmed cell death - insights from *Blastocystis* deathstyles | Observation of autophagic-like vacuoles by TEM, suggestive of non-apoptotic programmed cell death |
| Tan and Suresh, 2006 | Predominance of amoeboid forms of *Blastocystis* *hominis* in isolates from symptomatic patients | Observation via TEM of two types of amoeboid form: one with electron-dense granules and a thin surface coat; the other with multiple small vacuole. The authors not that bacteria were observed to be frequently in close proximity to pseudopodia. |
| Yoshikawa et al., 2007 | Ultrastructural and Phylogenetic Studies on *Blastocystis* Isolates from Cockroaches | Isolates of *Blastocystis* from cockroaches were imaged using TEM and were found to be morphologically indistinguishable from *Blastocystis* isolates from other animals. |
| Zhang et al., 2012 | Ultrastructural insights into morphology and reproductive mode of *Blastocystis hominis* | Both SEM and TEM were utilised to reveal the ultrastructure of different morphological forms of *Blastocystis,* including vacuole, granular and multivacuolar forms. SEM found evidence of bacteria on the surface of *Blastocystis*. |
| Dhurga et al., 2016 | Granular Formation during Apoptosis in *Blastocystis* sp. Exposed to Metronidazole (MTZ) | Imaging showed the difference between *Blastocystis* treated with metronidazole and untreated *Blastocystis.* |
| Raman et al., 2016 | Increase number of mitochondrion-like organelle in symptomatic *Blastocystis* subtype 3 due to metronidazole treatment | TEM and SEM were performed on *Blastocystis* cells from both symptomatic and asymptomatic subject, exposed to, or not exposed to metronidazole |
| Thergarajan et al., 2018 | *In vitro* and *in vivo* thermal stress induces proliferation of *Blastocystis* sp. | TEM imaging of Blastocystis cultures grown at 37°C and cultures thermal stressed at 41°C, which contained many granules within the central body, and fewer vacuoles. |

References:

Chen, X.Q., Singh, M., Howe, J., Ho, L.C., Tan, S.W., Yap, E.H., 1999. *In vitro* encystation and excystation of *Blastocystis ratti*. Parasitology 118, 151–160. https://doi.org/10.1017/S0031182098003667

Dhurga, D.B., Suresh, K., Tan, T.C., 2016. Granular formation during apoptosis in *Blastocystis* sp. exposed to metronidazole (MTZ). PLoS One 11, e0155390. <https://doi.org/10.1371/journal.pone.0155390>

Duda, A., Stenzel, D.J., Boreham, P.F.L., 1998. Detection of *Blastocystis* sp. in domestic dogs and cats. Vet Parasitol 76, 9–17. <https://doi.org/10.1016/S0304-4017(97)00224-0>

Dunn, L.A., Boreham, P.F.L., Stenzel, D.J., 1989. Ultrastructural variation of *Blastocystis* *hominis* stocks in culture. Int J Parasitol 19, 43–56. https://doi.org/10.1016/0020-7519(89)90020-9

Lee, M.G., Stenzel, D.J., 1999. A survey of *Blastocystis* in domestic chickens. Parasitol Res 85, 109–117. https://doi.org/10.1007/s004360050518

Mehlhorn, H., 1988. *Blastocystis hominis*, Brumpt 1912: Are there different stages or species? Parasitol Res 74, 393–395. <https://doi.org/10.1007/BF00539464>

Moe, K.T., Singh, M., Howe, J., Ho, L.C., Tan, S.W., Chen, X.Q., Yap, E.H., 1999. Development of *Blastocystis hominis* cysts into vacuolar forms *in vitro*. Parasitol Res 85, 103–108. https://doi.org/10.1007/s004360050517

Moe, K.T., Singh, M., Howe, J., Ho, L.C., Tan, S.W., Ng, G.C., Chen, X.Q., Yap, E.H., 1996. Observations on the ultrastructure and viability of the cystic stage of *Blastocystis hominis* from human feces. Parasitol Res 82, 439–444. https://doi.org/10.1007/s004360050142

Nasirudeen, A.M.A., Hian, Y.E., Singh, M., Tan, K.S.W., 2004. Metronidazole induces programmed cell death in the protozoan parasite *Blastocystis hominis*. Microbiology (N Y) 150, 33–43. https://doi.org/10.1099/mic.0.26496-0

Nasirudeen, A.M.A., Tan, K.S.W., 2004. Isolation and characterization of the mitochondrion-like organelle from *Blastocystis hominis*. J Microbiol Methods 58, 101–109. https://doi.org/10.1016/j.mimet.2004.03.008

Nasirudeen, A.M.A., Tan, K.S.W., Singh, M., Yap, E.H., 2001. Programmed cell death in a human intestinal parasite, *Blastocystis hominis*. Parasitology 123, 235–246. <https://doi.org/10.1017/S0031182001008332>

Raman, K., Kumar, S., Chye, T.T., 2016. Increase number of mitochondrion-like organelle in symptomatic *Blastocystis* subtype 3 due to metronidazole treatment. Parasitol Res 115, 391–396. https://doi.org/10.1007/s00436-015-4760-0

Singh, M., Suresh, K., Ho, L.C., Ng, G.C., Yap, E.H., 1995. Elucidation of the life cycle of the intestinal protozoan *Blastocystis hominis*. Parasitol Res 81, 446–450. https://doi.org/10.1007/BF00931510

Stenzel, D.J., Boreham, P.F., 1996. *Blastocystis hominis* revisited. Clin Microbiol Rev 9, 563–584. https://doi.org/10.1128/CMR.9.4.563

Stenzel, D.J., Cassidy, M.F., Boreham, P.F.L., 1994. Morphology of *Blastocystis* sp. from domestic birds. Parasitol Res 80, 131–137. https://doi.org/10.1007/BF00933780

Stenzel, D.J., Cassidy, M.F., Boreham, P.F.L., 1993. Morphology of *Blastocystis* sp. isolated from circus animals. Int J Parasitol 23, 685–687. https://doi.org/10.1016/0020-7519(93)90179-3

Stenzel, D.J., Lee, M.G., Boreham, P.F.L., 1997. Morphological differences in *Blastocystis* cysts - an indication of different species? Parasitol Res 83, 452–457. <https://doi.org/10.1007/s004360050279>

Suresh, K., Chong, S.Y., Howe, J., Ho, L.C., Ng, G.C., Yap, E.H., Singh, M., 1995. Tubulovesicular elements in *Blastocystis hominis* from the caecum of experimentally-infected rats. Int J Parasitol 25, 123–126. https://doi.org/10.1016/0020-7519(94)00074-X

Tan, H.K., Harrison, M., Zierdt, C.H., 1974. Freeze-etch studies of the granular and vacuolated forms of *Blastocystis hominis.* Zeitschrift für Parasitenkunde 44, 267–278. https://doi.org/10.1007/BF00366110

Tan, H.K., Zierdt, C.H., 1973. Ultrastructure of *Blastocystis hominis*. Zeitschrift für Parasitenkunde 42, 315–324. https://doi.org/10.1007/BF00328892

Tan, K.S.W., Howe, J., Yap, E.H., Singh, M., 2001. Do *Blastocystis hominis* colony forms undergo programmed cell death? Parasitol Res 87, 362–367. https://doi.org/10.1007/s004360000364

Tan, K.S.W., Nasirudeen, A.M.A., 2005. Protozoan programmed cell death – insights from *Blastocystis* deathstyles. Trends Parasitol 21, 547–550. <https://doi.org/10.1016/j.pt.2005.09.006>

Tan, S.W., Singh, M., Ho, L.C., Howe, J., Moe, K.T., Chen, X.Q., Ng, G.C., Yap, E.H., 1997. Survival of *Blastocystis hominis* clones after exposure to a cytotoxic monoclonal antibody. Int J Parasitol 27, 947–954. https://doi.org/10.1016/S0020-7519(97)00066-0

Thergarajan, G., Govind, S.K., Bhassu, S., 2018. *In vitro* and *in vivo* thermal stress induces proliferation of *Blastocystis* sp. Parasitol Res 117, 177–187. https://doi.org/10.1007/s00436-017-5688-3

Yoshikawa, H., 1996. Freeze-fracture cytochemistry of membrane cholesterol in *Blastocystis hominis*. Int J Parasitol 26, 1111–1114. https://doi.org/10.1016/S0020-7519(96)00091-4

Yoshikawa, H., Kuwayama, N., Enose, Y., 1995. Histochemical detection of carbohydrates of *Blastocystis hominis*. Journal of Eukaryotic Microbiology 42, 70–74. https://doi.org/10.1111/j.1550-7408.1995.tb01542.x

Yoshikawa, H., Morimoto, K., Nagashima, M., Miyamoto, N., 2004. A survey of *Blastocystis* infection in anuran and urodele amphibians. Vet Parasitol 122, 91–102. https://doi.org/10.1016/j.vetpar.2004.03.018

Yoshikawa, H., Wu, Z., Howe, J., Hashimoto, T., Geok-Choo, N., Tan, K.S.W., 2007. Ultrastructural and phylogenetic studies on *Blastocystis* isolates from cockroaches. Journal of Eukaryotic Microbiology 54, 33–37. <https://doi.org/10.1111/j.1550-7408.2006.00141.x>

Zhang, X., Zhang, S., Qiao, J., Wu, X., Zhao, L., Liu, Y., Fan, X., 2012. Ultrastructural insights into morphology and reproductive mode of *Blastocystis hominis*. Parasitol Res 110, 1165–1172. <https://doi.org/10.1007/s00436-011-2607-x>

Zierdt, C.H., 1986. Cytochrome‐free mitochondria of an anaerobic protozoan— *Blastocystis hominis*. J Protozool 33, 67–69. https://doi.org/10.1111/j.1550-7408.1986.tb05559.x
